# Perfectionism moderates the relationship between drive for thinness and exercise dependence in Anorexia Nervosa

**DOI:** 10.1192/j.eurpsy.2026.12032

**Published:** 2026-07-31

**Authors:** M. De Stefano, C. La Femina, M. Di Mauro, R. Pecoraro, R. Ceres, G. Cascino

**Affiliations:** Università degli Studi di Salerno, Salerno, Italy

## Abstract

**Introduction:**

Excessive practice of physical exercise is a frequent symptom of Anorexia Nervosa (AN). The cognitive-behavioral model of compulsive exercise identified key constructs operating in the maintenance of exercise in people with AN, such as the eating psychopathology and perfectionism. Indeed, exercise is undertaken primarily to change weight or shape, and perfectionism is a robust correlate of excessive exercise. Although an integration of key factors into a theoretically coherent conceptualization has been proposed, the relationships between those factors has not been tested.

**Objectives:**

Aim of the present study was to investigate the relationship between drive for thinness, perfectionism and exercise dependence in AN. We hypothesized that perfectionism could moderate the relationship between eating psychopathology and exercise dependence.

**Methods:**

A total of 102 women with AN completed the Exercise Dependence Scale – Revised (EDS-R) and the Drive for Thinness and Perfectionism subscales of EDI-2. A simple moderation model with the effect of drive for thinness on exercise dependence moderated by the levels of perfectionism was performed.

**Results:**

The interaction between drive for thinness and perfectionism in relation to exercise dependence was significant (*b* = 0.17; *p* = 0.03; CI 0.44, 10.72). A simple slopes analysis was performed to assess the interaction in terms of ranges of perfectionism (-1SD and +1SD) where drive for thinness is related to exercise dependence. The relationship between drive for thinness and exercise dependence was significant for both low and high levels of perfectionism (*b* = 0.31, *p* = 0.001), (*b* = 0.64, *p* < 0.001), with a higher slope at higher levels of perfectionism.

**Conclusions:**

These findings show the interplay between eating pathology and perfectionism as operating in the maintenance of excessive and inappropriate physical exercise, one of the most frequent symptoms of AN. This interaction might have psychotherapeutic implications, since targeting the dimensions of perfectionism (i.e. high personal standards and tendency towards self-criticism) could mitigate the association between eating psychopathology and exercise engagement.

**Disclosure of Interest:**

None Declared

